# A study on psychological stress assessment of higher educational institution’s students based on computer data mining technology

**DOI:** 10.3389/fpsyg.2022.1054907

**Published:** 2023-01-05

**Authors:** Shaohong Chen

**Affiliations:** Institute of Foreign Languages, Guangzhou Huashang College, Guangzhou, Guangdong, China

**Keywords:** psychological stress, HEI’s, pressure sensor technology, data mining, China

## Abstract

Safety begins in the human mind, and the individual’s need for psychological safety is the most fundamental need. This fundamental need takes psychological safety as the starting point for the gradual formation of a connection with the outside world. Graduates are the backbone of China’s economic development, and their healthy development is of great significance to the sustainable development of China’s economy and society. Excessive psychological pressure may bring pain to students in physical and psychological aspects, and even lead to suicide. If students with abnormal psychological pressure can be found in time, the school can provide help and intervention in time to relieve psychological pressure. This paper uses pressure sensor technology for data collection and data mining techniques for data analysis to assess and predict the psychological stress level of Higher Educational Institutions (HEIs) students. The results show that this method can accurately and objectively evaluate the psychological stress of the students, and the evaluation results of students’ psychological stress are stable, which can provide students with psychological stress assessment services. It implies that HEI’s policymakers should consider these techniques to assess the psychological stress of the students proactively.

## 1. Introduction

With the emergence of various social forms, the state of mind of today’s HEIs graduates has changed a lot compared with that of the past. The present living conditions, family background, and academic performance, etc. have led to the cause of various negative psychological emotions in the HEIs students, among which melancholy and anxiety are the most prominent (Seggie et al., 1974).

Stress is an external imbalance that stimulates the body when the external danger does not eliminate, causing physical and mental damage that can lead to the destruction of life. Lazarus, an American psychologist, states that stress is an external demand beyond what the individual, the social system, or the body’s organizational system can bear (Daoyang, 2004). Once a negative psychological state occurs, it can negatively impact their future studies and work (Chaomin, 2014). These people may be unaware of their place in the circle and have difficulty adapting, creating a huge obstacle to their future development. That would be a huge blow, both to their families and to the country as a whole (Yao and Donglin, 2022).

With the development of science and technology, people’s living standards have improved significantly. However, this is accompanied by many mental problems (Ho-Young and Lan, 2001), especially among college students. When a senior high school freshman enters the campus, he faces different life from the past, and many college students feel unable to adapt to this new learning style (Shihua et al., 2021). In addition, the relationship around him is changing, and he must get to know more people. When he leaves home for the first time, he must solve all problems by himself. The first-grade students will have negative psychological feelings, such as depression, anxiety, and loneliness (Rice and Lin, 2000), which will lead to a loss of interest in learning and not wanting to communicate with others. For students, appropriate pressure can improve their learning efficiency, which is conducive to their growth and development. However, excessive psychological stress may bring pain to students in physical and psychological aspects, and even lead to suicide. If students with abnormal psychological stress get recognized in time, the school can provide help and intervention timely to relieve psychological stress.

At present, regular questionnaires assess psychological stress mainly. However, this method can only reflect the psychological pressure of the students participating in the survey at that time, and cannot obtain the pressure state of the students not participating in the survey. In addition, students may be perfunctory when filling in the questionnaire, or intentionally fill in wrong information in order to conceal the truth. Therefore, it is the need of time to use some recently emerged techniques to unearth the actual level of psychological stress that students face during their stay at HEIs.

Data mining technology is developed from statistics, databases, and machine learning. It is a process of extracting information and knowledge that people do not know yet, but is useful for the potential decision-making process from big data, and these data and knowledge can provide services for educators, learners, managers, educational software developers, and educational researchers (Ting and Gangshan, 2010). For example, the identification of poor students (Hengxin et al., 2009), analysis of students’ online behavior (Shuai et al., 2016), and academic early warning of students and employment prediction can be done through data mining (Campagni et al., 2015; Xing et al., 2015). Data collection is generally obtained through interviews, questionnaires, students’ daily behaviors, and teaching resources. This study uses sensor data acquisition. Sensor module is built on the surface of the sensor, on the parameters of the environment that produce changes in electrical parameters of sensitive devices, such as changes in temperature impact on the resistance value of the thermistor, changes in humidity impact on the resistance value of the moisture sensitive resistor, changes in the brightness of the light impact on the resistance value of the photoresistor, and so on. The difference between changes in the need for an AD digital-to-analog conversion module is used to obtain the small environmental parameters caused by changes in electrical parameters through the signal processing parameters to filter out the wrong data, and the amplification circuit can be helpful to obtain a quantifiable electrical parameter. It leads the microcontroller to identify the electrical parameters and complete the acquisition of environmental data. Using the sensor, the analog amount of information is converted into an electrical signal output, and the main controller makes judgments and conversions to derive the collected analog amount of information. This design uses the characteristics of the sensor to convert pressure signal changes into electrical signal output.

Focusing on the mentioned techniques that have emerged recently, the present study is unique that it employs data mining technology. It uses students’ personal information, daily campus life data, and data collected from students’ responses to the stress assessment questions to ascertain the psychological stress level of the students. This study will ultimately help to conduct timely counseling of the students for their better future.

The rest of the paper is structured as follows: section two presents the recent literature on students’ psychological stress and the use of data mining techniques; section three describes the research methodology employed in this research; section four shows the results of statistical analysis; and final section concludes the study with necessary policy implications and future research directions. The following picture 1 shows the implementation steps of this study.

## 2. Literature review

Graduates of higher education are the most important talents of the country. Only with good psychological quality can they study hard and contribute to national construction (Zhang et al., 2003). In recent years, the psychological problems of Chinese college students have become increasingly noticeable. The mental issues of university students have become increasingly prominent, and the students are being educated and discussed in depth (Danqing, 2021). Among the mental issues of university students, anxiety and depression are the most significant and harmful factors (Yang et al., 2006). Previous psychological surveys have shown that the number of university students with psychological problems in the country is no longer a small percentage, accounting for 16–25% of the national total. Every year dozens of students finish their lives due to relationship problems, failed relationships, failed exams, and unsatisfactory lives (Zhang, 1994). A study conducted in Shanghai found that 20% of the 200 unhappy factors were confused about the future, relationship management, and problems with the opposite sex (Xiting, 1997).

Indeed, there are many different definitions of stress; different researchers have different views and perceptions about the definition of stress due to different purposes and methods of research: some equate stress with stressful events (Hong and Jinrong, 2002), stressful stimuli, and stressors, such as Brown who states that stress is an event that may endanger an individual or hinder the individual’s response. Selye (2015) believes that stress is ubiquitous in life, and everything that happens in one’s life is likely to become a source of stress. Basudan et al. (2017) believe that stress is a disease caused by external stimuli, by some external physiological or psychological factors, which affect the physical and mental health of individuals. The research of Sulkowski et al. (2011) shows that stress can lead to overeating, and a long-term stress state is full of risks to mental and physical health. Lin Chun-mei, on the other hand, treats stress as psychological pressure and psychological stress. Chun-mei (2001) states “Psychological stress is stress that is caused by the body’s reaction to internal and external conditions and the influence of external factors.” Xiao-feng (2013) regards pressure as a relationship between people and the environment, which requires individuals to deal with it. According to Chang (2007), mental stress is a feeling of overwhelm, a feeling that cannot be dispelled for the time being. Others have considered various kinds of stress from both internal and external perspectives in an attempt to paint an exhaustive picture of it. Hee-ting (1991) divides stress into three main categories: stressors, stress reactions, and stress feelings. Shin Ho-young et al. classify stress into tension, stress, and various activities that trigger stress (Linxian, 2007).

The traditional assessment method of students’ psychological stress is mainly realized through students’ self-assessment and manual interview (Hui, 2011). The self-assessment method is mainly used by students to fill in the self-assessment scale to measure the source of psychological pressure and the state of psychological pressure, which is the most widely used method at present. However, this method can only reflect the psychological pressure of students who participated in the survey and cannot obtain the stress state of students who did not participate. In addition, students may be perfunctory or intentionally fill in wrong information when filling in the questionnaire, and the true state of psychological stress will be difficult to find. The manual interview needs to evaluate the psychological pressure of students through daily or multiple interviews lasting for a period of time (Lee and Hong, 2018). This method usually takes a long time and only works for some students who are active and cooperative. By mining students’ personal information and pressure sensor data, this research evaluates students’ psychological stress state in a passive way. The use of the established model can be extended to every student at HEIs.

## 3. Research design

### 3.1. Statistical technique

This paper used a data mining technique to achieve the objectives of the study. Data mining involves many methods, and the selection of appropriate methods depends on the data type and the specific purpose of data analysis (Yang et al., 2005). Data mining can be divided into various types, such as classification learning, supervised learning, clustering analysis, association rule mining, prediction mining, time series mining, and deviation analysis (Xiang, 2019). In addition, the internationally known brain functional imaging technology provides strong support for college students’ emotional problems (Yihan et al., 2022). Many researchers have analyzed brain imaging data to find the exact location of the brain-activated area of students suffering from depression, anxiety, and other negative emotions (Jing, 2020). Several researchers are now focusing on the neutron’s stationary state. By calculating the resting state, we can understand the brain activity of students with negative emotions when they are quietest (Wenyi and Hao, 2022). In this way, doctors can use the images of brain functions to understand their psychological state and to make a correct diagnosis (Compas et al., 1985).

**Figure 1:**
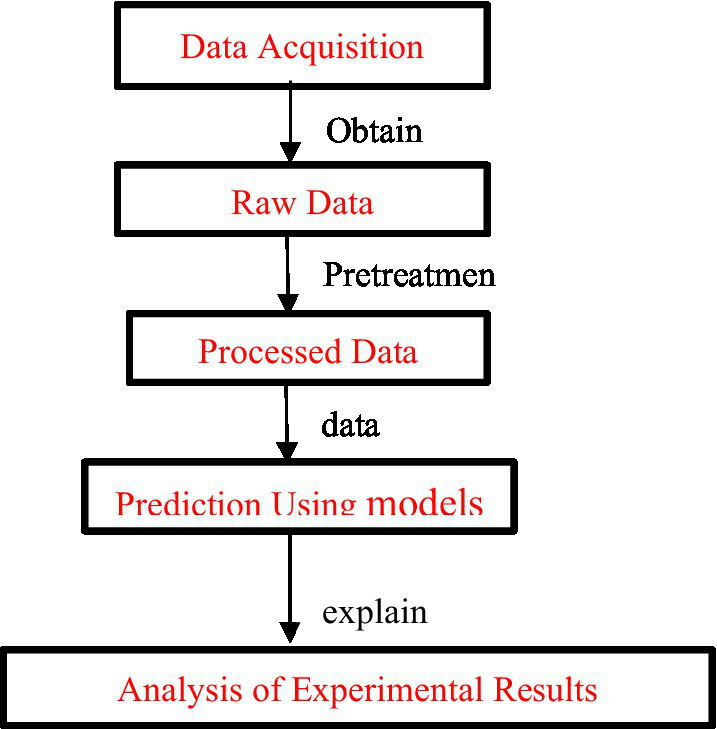
Steps of data mining.

Over the past few years of continuous exploration and research, a process has been developed that is the basis for data collection techniques. It consists of extracting and transforming the required data from uncleaned raw data, building a pattern of classifiers or clusters on top of this, and then extracting and processing the data (Yingqiu and Yali, 2008). The flowchart is shown in Figure 1.

The present research first uses sensor data about the stress intensity of students’ responses to stress assessment questions, then extracts students’ necessary information from the school database and carries out the standardized conversion of the data. Afterward, it cleans the incorrect, abnormal, and inconsistent data. Then, it loads the data into the warehouse structure. Finally, it uses Microsoft SQL Server Analysis Services for data mining and creates four different data mining models to get the best results for the study.

#### 3.1.1. Sensor data collection

These data acquisition is carried out using pressure sensors to collect data on the response pressure strength of students answering questions on a pressure assessment. Working principle: the finger is bound to the press module during the assessment, and data are collected on its response strength during the answer time, so that the sliding rheostat at the set position of the detection module acts to send out a pressure signal and the pressure signal felt is transmitted to the ADC0832 conversion chip by converting it into a voltage signal, which is converted into a digital signal by A/D conversion and transmitted to the microcontroller, which converts the current collected pressure signal according to the input signal. The microcontroller converts the currently collected pressure signal according to the input signal and finally transfers the collected pressure signal each time to the database for storage. The data can be analyzed according to the different signal stimulation intensities and the assessment answer scores.

#### 3.1.2. Data extraction

Because there are large amount of data in the database covering various fields, but not all of it is necessary for data warehousing, different kinds of data have to be extracted from the database according to the needs of data warehousing. Considered from a data warehousing point of view, not all data need to be run in a database. In general, the data warehouse will extract the necessary information from the analysis of the requirements. Firstly, the selection is based on the actual situation of the user. Depending on the user’s concerns, the information selected will vary. For example, the school administration can only count the scores of students while the school’s admissions office can only investigate the personal data of students (Honglin, 2006). Secondly, the relevant information is extracted based on a specific topic. The method can be a combination of manual, semi-automatic, or automatic methods (Xinhuan, 2014).

#### 3.1.3. Data transformation

Data conversion is also a necessary part of establishing data warehousing process. In order to prevent inconsistent information due to differences in business, it is important to standardize the various data in the database. There are inconsistencies in various existing databases, such as IBMDB2, Oracle, SQL Server, Excel, etc. There are also many cross-cutting issues in real life (Xiaochun, 2011).

As there is a large amount of information in Excel forms in various formats, the information in the same field can also be very different and, therefore, must be converted.

#### 3.1.4. Data cleaning

The accuracy of data is essential in data mining techniques as it is key to its precision.

To obtain accurate information, multiple sources of information must be cleaned. In this context, “cleaning” refers to the correction or removal of incorrect or inconsistent data when it is entered into a database to avoid adverse effects on the correct judgment of the decision support system (Yihan et al., 2022). That is Qiang and Baojuan (2014), when data are transformed, incorrect data, abnormal data, and inconsistent data must be removed and transformed, data gaps must be filled, isolated locations in the data set identified, and interfering data and data inconsistencies excluded. Some common ways of doing this are as follows:

1. Removing this data record.
2. Relying on the direct human experience.
3. Filling in blank values.

Because the focus of this system is on psychometric tests for university students and specifically on psychometric test scores, most of the data ensure the integrity of the test.

#### 3.1.5. Data loading

The task of data loading is to load all the data according to the already designed data into the warehouse structure (Guangyuan et al., 2013).

The main steps include space-filling and validity checking. For this system, the raw data formats are diverse. Since 2001, when the Department of Educational Technology at the University of X used information technology to store results, there have been three changes in the format of the data handled by the teachers of the various subjects. The early method of aggregating the data was outdated, storing all the Excel tables (one for each course) in different folders by date, which also contained data from other departments, therefore, required special filtering, which is a very time-consuming process. Instead of using a special data SSIS tool to extract and load the data, a special data extraction program was written using Visual Studio to process the data (Wei et al., 2014).

## 4. Statistical analysis and results

### 4.1. Overview of analysis implementation

This research uses Microsoft SQL Server Analysis Services for data mining and pressure sensor technology for data collection. Because it is not known which data mining method will be used to get the closest results to the actual situation, we will create four different data mining models to select the best results for the study. Also, we have chosen data from 2022 to predict because in 2022, all students completed the psychological stress test; they also got an average to see the difference between the real and the expected (Yanhong, 2013).

### 4.2. Sensor data communication preparation

In this design, a serial module is used to establish a communication network between the main control chip and the upper computer, enabling the uploading of three-way pressure detection data, which can help in informing the user of the current transformer situation promptly. The main chip of the module is the CH340G, which is used more frequently in various communication systems, especially in the Internet of Things (IoT), and can meet the requirements of long-distance transmission with low transmission delay to ensure the quality of data communication. The CH340G is capable of emulating a standard serial port, allowing serial port operation, and is fully compatible with the vast majority of original serial port applications. The role of the serial chip is to convert the TTL level output by the microcontroller to a 232 level that the PC can receive or to convert the 232 level output by the PC to a TTL level that the microcontroller can receive.

**Figure 2:**
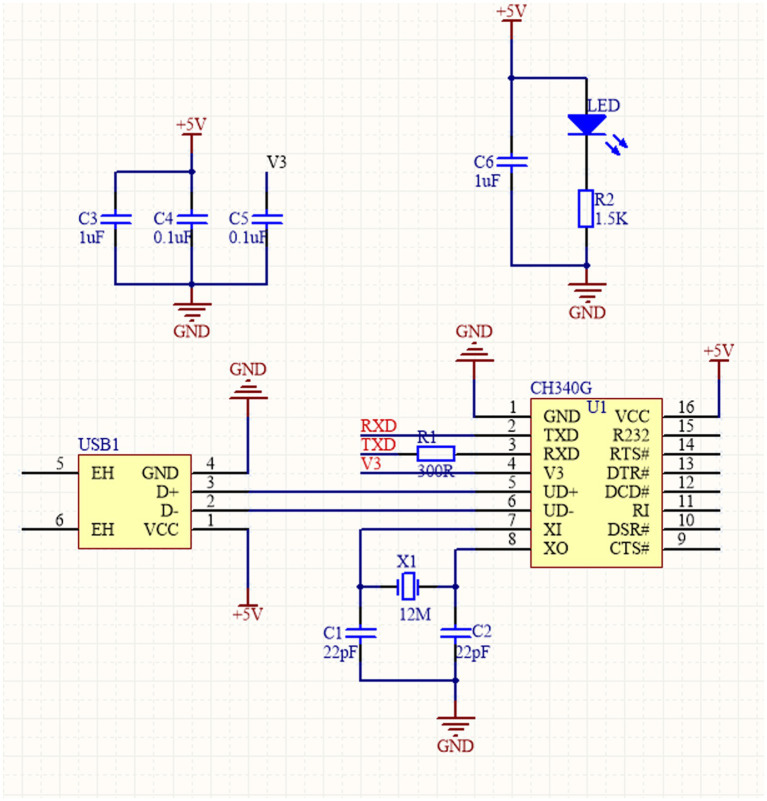
Sensor data communication module circuit.

The CH340G module communicates with the master chip *via* a serial port for data communication. The RX and TX pins of the CH340G chip are connected to the TX and RX pins of the microcontroller, respectively. The serial communication module circuit is shown in Figure 2.

**Figure 3:**
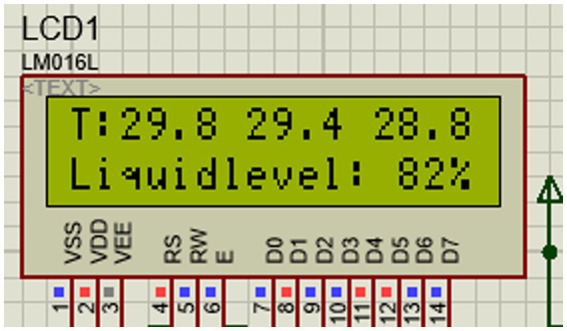
LCD interface diagram.

After access to communication, press the debugging button, and the LCD will display the three-way pressure value and pressure value in real-time. It can be observed that the first line of the liquid crystal shows the pressure value A of the three sensors, and the second line shows the pressure value B. The LCD interface is shown in Figure 3 below.

The microcontroller drives the serial port to transmit the collected reaction pressure data to the upper computer. Open the upper computer to set the serial port connection. After setting the baud rate and port, click “Connect,” and you can observe that the connection is successful. After completion, the reaction pressure data can be observed on the interface of the serial port assistant, and the value change is synchronized with the value on the LCD to realize the function of remote data collection.

### 4.3. Data mining preparation

The first step using the SQL Server Analysis service, create the project for the analysis service for Data Mining.sln. Create a new source in this project, link it to the previous data store, and then create a source view to join the existing source view.

Using the 2019–2022 view as a set of training materials, the 2022 (Year 1 to Year 2 scores) view and the 2022 (Year 1 to Lower Sophomore scores) view (already known), as described earlier (Chen et al., 2011).

The second step is called excavation construction. It is a must to first identify the input and forecast rows for the data mining technique to apply to the training material; on this basis, the correspondence between the input and forecast columns must be established. A mathematical model for data mining was built using an example of the decision tree algorithm, setting a forecast level mean; the input column was set to psychometric test score; in addition, the value of the keyword was set to “school number.” Based on this, the association between the input and the forecast was analyzed using data mining methods, and a valuable forecast was made.

Step three involves adding a pattern to the data collection. Right-click under the Mining Patterns tab and select a new mining pattern to add a decision tree algorithm, a cluster analysis algorithm, a neural network algorithm, and a logistic regression algorithm. Use the same data collection architecture.

### 4.4. Analysis of the accuracy of mining models

A single algorithm cannot be used for specific data mining. However, the same data can be used for some specific data mining, so biased speculation before the final result appears can easily lead to inaccurate conclusions. For this reason, the authors use different data mining methods to analyze and predict the available data and finally arrive at the optimal method.

**Figure 4:**
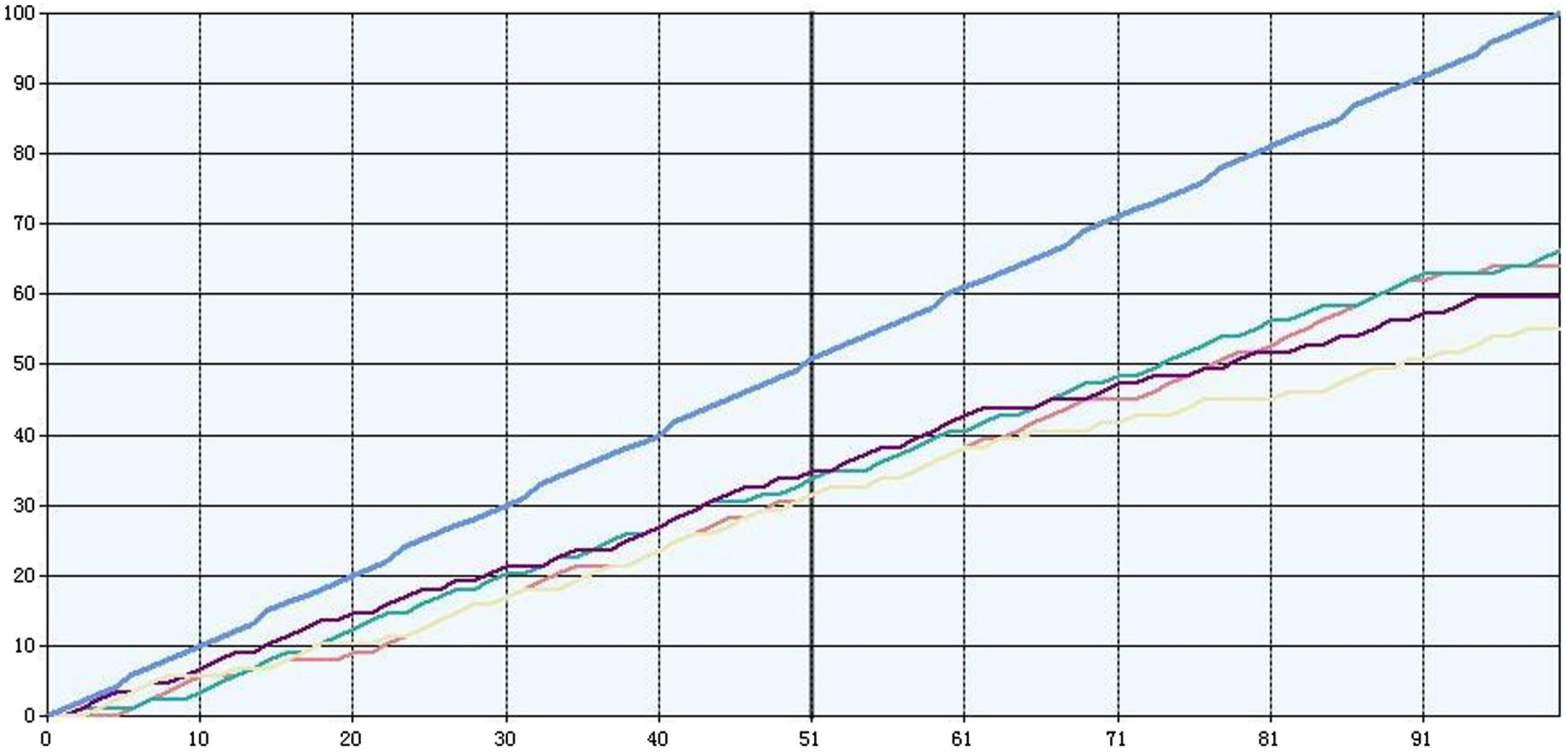
Lifting diagram.

In this paper, the data mining accuracy curves in the Microsoft SQL Server Analysis service are used to compare the four patterns, i.e., to observe the performance of the four patterns when performing the same data architecture. Proceed to Figure 4 to get its data.

The blue diagonal line is the ideal model and is the baseline against which the lift is assessed. The other four different colored lines indicate the performance of the four different models in terms of prediction.

SSAS compares these four models on several dimensions, as shown in Table 1.

**Table 1:** Comparison of different models.

Sequences, models	Score	Overall correct	Predicted probability
Logistic regression algorithms	0.63	31.46%	74.98%
Neural network algorithms	0.66	33.71%	77.24%
Decision tree algorithms	0.66	34.83%	53.32%
Cluster analysis algorithms	0.58	31.46%	58.60%
Ideal models		51.00%	

Within these indicators, “scores” allow for a holistic approach to comparing the validity of models across criteria. The higher the score, the better the model. The value of the “forecast probability” represents the threshold that the customer would need in a “likely to buy” situation. In each instance, the model estimates the accuracy of each forecast and stores it to select the most accurate data mining method. From the above data, it is easy to see that of the four models, neural network has the greatest chance of predicting 77.24%, hence the choice of the neural network in this paper.

### 4.5. Prediction using models

On this basis, we will use the neural network roadshow algorithm to predict the data. The above information is practiced using this method, and then we will introduce forecast data to predict the average test result. The methods are:

1. Switch to the prediction tab of mining mode, click the “Mode” button, and select “Most Accurate Data Mining Neural Network” in the pop-up dialog box;
2. In the input table on the right, select Normal_2022 (in the sophomore year), so that the learning mode on the left and the fields of the input table will be.
3. The information to be displayed in the final results are given in Table 2.

**Table 2:** Structure of the predicted results.

Source	Field	Conditions/parameters
normal_2022(Sophomore on)	Student number	None
normal_2022(Sophomore on)	Class Id	None
normal_2022(Sophomore on)	Sex	None
normal_2022(Sophomore on)	Average psychometric test scores (actual values)	None
Neural network algorithms	Average psychometric test scores (predicted)	None
Prediction functions	Predict probability	[Neural network algorithm]. [Predicted values].

Firstly, there is the base information, which contains the number of credits, class, and gender, and then the base values for the class of 2022, as well as the predicted performance values using the neural network and the probability of the predicted grades determined by the method. Thus, using the method, it is possible to analyze the data for the class of 2022 and make predictions about their final test scores, as it is still in the experimental stage and not yet official. We can judge the accuracy and reliability of the data by making predictions about the average test scores of the students in 2022.

The normal_2022 (under sophomore) view (under sophomore) was predicted, and their scores on the psychological stress test up to a sophomore year and their scores on the test from freshman to under sophomore year were compared.

### 4.6. Study results

After setting the fields according to the requirements above and switching to the results option, the results of the data mining can then be seen and the results saved to SQL Server as follows.

1. Predict the results when the table is in the normal_2022 (sophomore) view, as shown in Table 3.

**Table 3:** Some results when the prediction table is normal_2022 (Sophomore upper).

Student number	Classes	Gender	Actual value	Predicted value	Prediction accuracy
80,113,436	202,201	Male	1.5	2	0.610708354
80,113,437	202,201	Male	3	3	0.377110042
80,113,441	202,201	Female	2.5	2.5	0.512550846
80,113,442	202,201	Female	2.5	1.5	0.290128239
80,113,443	202,201	Female	2.5	3	0.404747159
80,113,444	202,201	Female	2	2	0.503364539
80,113,445	202,201	Female	2.5	2	0.426135176
80,113,446	202,201	Female	2	2	0.350896618
80,113,447	202,201	Female	2.5	3	0.341418698
80,113,448	202,201	Female	2	2	0.545598651
80,113,449	202,201	Female	2	2.5	0.424083464
80,113,450	202,201	Female	2.5	3	0.485778161
80,113,451	202,201	Female	2	2	0.53151616
80,113,452	202,201	Female	3	2.5	0.478285234
80,113,453	202,201	Female	2.5	3	0.457179444
80,113,454	202,201	Female	2.5	2.5	0.374685875
80,113,455	202,201	Female	2.5	2	0.676034401
80,113,456	202,201	Female	2.5	3	0.365396023
80,113,457	202,201	Female	2	2	0.535056509
80,113,458	202,201	Female	2	2.5	0.402580328
80,113,459	202,201	Female	2.5	2.5	0.369597333

Since there are 40 predictions in total, only a small selection of them is listed here. From the table above, the students were analyzed for data and for he can see that by comparing the 202 examinations, we can see that the average of their scores is the same as their actual scores after comparison. After further processing the above information, the result of the query shows that a total of 17 data items meet the requirements.

2. The results of the prediction table for the normal_2022 (under sophomore year) view as shown in Table 4.

**Table 4:** Partial results for the prediction table for normal_2022 (under sophomore year).

Student number	Classes	Gender	Actual value	Predicted value	Prediction accuracy
80,113,436	202,201	Male	1.5	2	0.619432296
80,113,437	202,201	Male	3	3	0.645238327
80,113,441	202,201	Female	2.5	2.5	0.500794354
80,113,442	202,201	Female	2.5	3	0.629051287
80,113,443	202,201	Female	2.5	3	0.650299022
80,113,444	202,201	Female	2	2	0.620749318
80,113,445	202,201	Female	2.5	3	0.453388769
80,113,446	202,201	Female	2	2	0.424878951
80,113,447	202,201	Female	2.5	3	0.627354405
80,113,448	202,201	Female	2	2	0.528929664
80,113,449	202,201	Female	2	3	0.599644427
80,113,450	202,201	Female	2.5	3	0.516437026
80,113,451	202,201	Female	2	2	0.579014835
80,113,452	202,201	Female	3	3	0.633618033
80,113,453	202,201	Female	2.5	2	0.366215011
80,113,454	202,201	Female	2.5	3	0.536548189
80,113,455	202,201	Female	2.5	2	0.381811887
80,113,456	202,201	Female	2.5	3	0.606786119
80,113,457	202,201	Female	2	3	0.38950895
80,113,458	202,201	Female	2	2.5	0.344746506
80,113,459	202,201	Female	2.5	3	0.423091955

Unlike the previous prediction result, the data in this prediction table contain more psychological stress test scores Therefore, the prediction is theoretically more accurate than the case where the prediction table is normal_2022 (upper sophomore year). In practice, the comparison shows that the final prediction accuracy is the same for both, but the prediction table for stress test scores also outperforms the prediction table for less stress test scores. For example, the student with student number 80,113,442 has a predicted value of 1.5 in the first case and a predicted value of 3 in the second case, whereas his actual GPA value is 2.5, so the prediction in the second case is more accurate. Therefore, the prediction in the second case is more factual. In addition, comparing the results of the two scenarios, it was found that the latter was generally more accurate than the first scenario, which partly supports the inference that more stress test scores are more accurate.

## 5. Conclusion

With the increasing employment pressure, social life challenges are increasingly severe, students’ academic pressures are also increasing, and it is easier to have a negative impact because of excessive psychological stress. The traditional method of psychological stress investigation is difficult to implement on a large scale due to complex operation, long duration, and other factors. If only a questionnaire is used to investigate, students may not take it seriously and can only wait for students to find a psychological center or counselor to master their psychological stress state. The evaluation through big data mining is a new mode that is more active, efficient, and can be widely implemented.

This paper has used data mining techniques to analyze the results of the quiz, both in theory and in practical application, and is limited by the technical aspects of the analysis, which can only be carried out through Microsoft’s internal data analysis. From the results above, we can see that the expected results can only be obtained if data mining methods are used. Thus, the present study uses pressure sensor technology for data collection and then data mining techniques for data analysis to assess and predict the psychological stress level of Higher Educational Institutions (HEIs) students. The results show that this method can accurately and objectively evaluate the psychological stress of the students, and the evaluation results of students’ psychological stress are stable, which can provide students with psychological stress assessment services. These findings have critical implications for the HEIs policymakers. First, it provides a base to consider these techniques in order to proactively assess the psychological stress of the students. Second, different colleges and universities can employ these techniques on the data of their students to check the psychological stress level among students and ascertain which data mining method is most suitable for their students. Last but not the least, a national-level policy can be proposed to use these emerging techniques at different levels of organizational staff to assess their level of stress during their job.

Maslow used the hierarchy theory to classify human needs into five levels. Among these needs, security is the most basic after physical needs. After the usual needs are secured, the higher-level needs of belonging, self-esteem, and self-actualization come into action. The more a person’s minimum requirements can be adequately met, the higher his needs will be. As Maslow said, when the needs for security and love are fulfilled, their frustration causes sickness. When analyzed from an individual perspective, there is still a significant proportion of people who do not feel sufficiently secure. Their security can make their lives more panicky and their mental health is inevitably adversely affected by the persistence of these problems. Therefore, it is important to fully recognize that psychological resilience plays a pivotal role in the psychological safety of university students, and enhancing their psychological safety through the active use and externalization of psychological resilience is the need of the hour.

With vast implications, this research has some limitations as well. First, the number of samples in this study is small, which has affected the training effect to a certain extent. If the number of participants can be increased, the model can have better results. Second, other algorithms can also be used in the future to increase the penalty of false judgment for samples, which should improve the effectiveness of the model.

In the future, this model can have the opportunity to be promoted in more colleges or schools through feasibility verification and can help students with psychological stress assessment services.

## Data availability statement

The original contributions presented in the study are included in the article/supplementary material, further inquiries can be directed to the corresponding author.

## Author contributions

SC: conceptualization, methodology, software, validation, formal analysis, resources, data curation, investigation, and writing—review and editing.

## Funding

This study was supported by the on campus program of Guangzhou Huashang College is “Research on the Psychological Stress Assessment of College Students Based on Data Mining Technology’’ (No.: 2022HSDS24).

## Conflict of interest

The author declares that the research was conducted in the absence of any commercial or financial relationships that could be construed as a potential conflict of interest.

## Publisher’s note

All claims expressed in this article are solely those of the authors and do not necessarily represent those of their affiliated organizations, or those of the publisher, the editors and the reviewers. Any product that may be evaluated in this article, or claim that may be made by its manufacturer, is not guaranteed or endorsed by the publisher.
